# Development and Performance of Negative Ion Functional Blended Yarns and Double-Sided Knitted Fabrics Based on ZnO/TM/PET Fiber

**DOI:** 10.3390/polym17070905

**Published:** 2025-03-27

**Authors:** Yingzi Zhang, Mengxin Zhang, Jishu Zhang, Jianbing Wu, Jiajia Peng

**Affiliations:** School of Textile Garment & Design, Changshu Institute of Technology, Changshu 215500, China; 201400004@cslg.edu.cn (Y.Z.); 20215215127@stu.suda.edu.cn (M.Z.); 201800012@cslg.edu.cn (J.W.); 202000031@cslg.edu.cn (J.P.)

**Keywords:** negative ion functional fiber, blended yarn, functional double-sided knitted fabric

## Abstract

Zinc oxide-modified tourmaline-based negative ion polyester fiber (ZnO/TM/PET), as a new functional fiber with excellent negative ion emission characteristics, is of great significance to human health, and its industrial application needs to be expanded and promoted. In this paper, using zinc oxide, tourmaline, and polyethylene terephthalate as the main raw materials, ZnO/TM/PET negative ion functional fiber with 5% ZnO/TM composites was prepared. Then, it was blended with cotton fiber and interknitted with wool yarn and spandex yarn, from which we developed five kinds of negative ion polyester/cotton-blended yarn and four different kinds of knitted double-sided fabric using different equipment and process parameters. The micromorphology of the fiber samples, the basic properties of the blended yarns, and the wearability and functional properties of the knitted fabrics were tested. The results show that the ZnO/TM negative ion additive is randomly dispersed in the polymer matrix without visible conglobation and the fiber has a good appearance. The blending ratio has an important effect on the properties of functional polyester/cotton blended yarn. The higher the ratio of negative ion polyester fiber in the blended yarn, the better the mechanical index of the blended yarn, the higher the negative ion emission, and the lower the hairiness index. The performances of fabric are influenced by the comprehensive action of fiber raw material type, yarn ratio, fabric tightness, and structure. The mechanical properties of the fabric knitted from negative ion polyester/cotton-blended yarn are lower than those made from negative ion polyester filament yarn. In the case of the same fabric structure, the negative ion emission performance, far-infrared emission performance, and antibacterial property of the fabric with a higher ratio of negative ion functional fiber is better than the lower ratio. With the same yarn composition, the negative ion emission performance and air permeability of the fabric with a loose structure are better than that of the fabric with a tight structure, but the moisture permeability, far-infrared emission properties, and antibacterial properties show little difference.

## 1. Introduction

Science and technology provide technical support for design, and design provides formal beauty for science and technology. The innovative integration of the two not only creates new fashion but also caters to the consumer behavior demands of today’s consumers. The invention of functional polymer fibers and their application in the textile field not only improves the scientific and technological content of textiles and added value of products but also improves people’s quality of life and environmental adaptability [1,2,3]. At present, the types of functional polymer fibers developed successfully can be roughly divided into four categories: basic functional fibers, bio-based functional fibers, high-performance functional fibers, and intelligent functional fibers. Basic functional fibers are functional fibers formed using nanotechnology, differentiation, composite, ultra-fine, copolymerization, blending, and grafting technologies to make polyester, acrylic, nylon, polypropylene, and other chemical synthetic fibers with various functions, such as hollow polyester functional cool fiber [4], graphene composite polyester fiber [5], high hygroscopic heating polyacrylonitrile fiber [6], ultra-fine polyethylene fiber [7], etc. Bio-based functional fibers are processed from agricultural, forest, and marine waste and by-products, whose raw materials are derived from renewable biomass, such as hydrophilic-modified chitosan bio-based fiber [8], fibroin bio-based fiber [9], skin-friendly bacteriostatic polylactic acid fiber [10], seaweed fiber [11,12], etc. High-performance functional fibers are chemical fibers with special physical and chemical structures, performances, and uses or special functions, such as colored polyimide fiber [13], polyether ether copper fiber [14], basalt fiber for protective gloves [15], high-elongation intermediate aramidon [16,17], etc. Intelligent functional fiber is a kind of fiber that can sense, process, and respond to changes in the external or internal environment such as temperature, light, and electricity, including phase-change storage adhesive smart fiber [18], temperature-sensitive and color-changing regenerative cellulose fiber [19,20], phase-change temperature-adjusted polypropylene fiber [21], etc. With the continuous changes in living environments, people’s functional needs for functional polymer fibers are becoming more and more diverse, and polymers with different functional forms are also continuously produced, which provides a richer source of raw materials for the development of new functional textiles.

Air-negative ions have special effects on the human body and are known as the “vitamin and growth hormone” in the air [22]. It can not only purify the air, remove odor, inhibit bacteria, disinfect, and reduce the harm of air pollution to the human body [23] but also improve the human lung capacity, balance blood pressure, calm sleep, and enhance the vitality of the human body, among other effects [24]. In order to develop negative ion functional fabrics with high negative ion emission, using ZnO/tourmaline (TM) composite material and PET chip as the main raw materials, our research group prepared a new type of negative ion functional fiber through the melt spinning process [25]. The fiber should belong to the basic functional fiber category in the functional fiber classification. Previous research results showed that the average particle size of the ZnO/TM composite is almost 365 nm, with an increase of nearly 50% in negative ion emission efficiency compared to the original TM. The apparent viscosity of the fiber masterbatch decreases with increases in the addition of ZnO/TM composite, and the rheological properties of PET fiber masterbatch are barely affected, which still shows shear thinning characteristics when the amount of addition reaches 10%. The ZnO/TM composite disperses well in the interior and surface of the ZnO/TM/PET fiber matrix. The prepared ZnO/TM/PET fiber has excellent properties, such as a fineness of 1.54 dtex, a fracture strength of 3.31 cN/dtex, and a negative ion release of 1640/cm^3^, which shows great industrialization potential. In the identification criteria of negative ion functional fabrics, the amount of negative ion generation is more than 1000/m^3^. It can be seen that the fiber has potential application prospects in textile, clothing, and household goods. However, the effects of spinning- and weaving-related process parameters on yarn and fabric properties are still unclear, and basic research on subsequent product development and industrial application is urgently needed.

In this paper, on the basis of previous research, using zinc oxide, tourmaline, and polyethylene terephthalate as the main raw materials, ZnO/TM/PET negative ion functional fiber with a 5% ZnO/TM composite was prepared. Then, it was blended with cotton fiber and interknitted with wool yarn and spandex yarn. Next, five kinds of negative ion polyester/cotton-blended yarn and four different kinds of knitted double-sided fabric using different equipment and process parameters were developed. The micromorphology of the ZnO/TM composite and ZnO/TM/PET fiber was observed, and the effects of the blending ratio on the performance of blended yarn and the yarn ratio and fabric structure on fabric wear and functional characteristics were analyzed, which provides an experimental basis and theoretical support for further promoting the industrial application of the anion functional polyester fiber.

## 2. Experiment

### 2.1. Experimental Materials

The main raw materials selected in this study are zinc oxide (Shanghai Macklin Biochemical Co., Ltd., Shanghai, China, 99.9% metals basis, 200 nm), tourmaline (Shijiazhuang Bo Rui Building Materials Co., Ltd., Shijiazhuang, China), polyester chip (Shanghai Thousand New Materials Co., Ltd., Shanghai, China), cotton fiber (Wuxi a cotton textile Group Co., Ltd., Wuxi, China), and spandex yarn (30 D, Zhejiang Huafon Spandex Co., Ltd., Wenzhou, China).

### 2.2. Preparation of ZnO/TM/PET Negative Ion Functional Fiber

**Figure 1:**
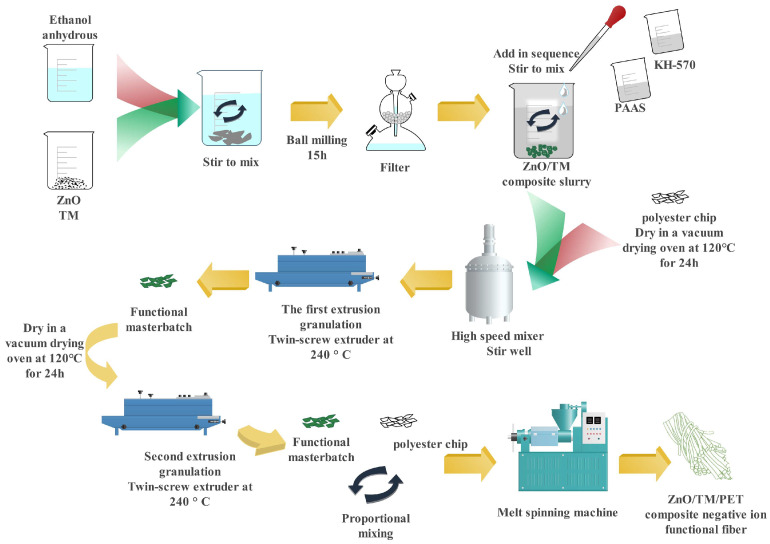
Process diagram of ZnO/TM/PET negative ion functional fiber.

Firstly, ZnO and TM were weighed according to a mass ratio of 1:2 and mixed with 10 mL of anhydrous ethanol. The mixture was placed in a stainless-steel ball grinding tank (a volume of 100 mL), where we set a ball mass ratio of 1:3, a ball mill speed of 300 RPM, and rotation directions of positive and negative rotation for 5 min, and then ground for 15 h to obtain the ZnO/TM composite slurry. The ZnO/TM composite slurry was continuously magnetically stirred for 2 h with KH-570 as the modifier and PAAS as the dispersant with a ratio of 18:0.8:1.8:80 (wt%). Polyester cuttings were dried at 120 °C for 24 h in a vacuum-drying oven and were then mixed evenly with the functional slurry in a high-speed stirrer. The mixture was extruded in a twin-screw extruder at 240 °C for the first time to produce functional pellets. Lastly, the functional pellets and polyester cuttings were evenly mixed in a high-speed stirrer and then dried for 48 h at 120 °C in a vacuum-drying oven. ZnO/TM/PET fiber with 5% ZnO/TM composites was prepared via the melt spinning mechanism. The whole process flow chart is shown in Figure 1. The temperature in each working area of the spinning equipment is shown in Table 1, and the process parameters are shown in Table 2.

### 2.3. Spinning of Negative Ion Polyester/Cotton-Blended Yarn

Consumers have increasingly diversified demands for clothing materials, hoping not only to have the comfort of natural fibers but also the durability and easy-care characteristics of synthetic fibers. Negative ion functional polyester and cotton-blended yarn can not only meet the diversified needs of consumers but also make full use of the advantages of the two kinds of fibers to improve the market competitiveness of products. In this part, five negative ion polyester/cotton-blended yarns were spun using miniature digital spinning equipment (Tianjin Huakai Jiacheng Technology Co., Ltd., Tianjin, China). The spinning equipment used includes an opening machine (SOp-04), a digital carding machine (DSCa-01), a digital drawing frame (DSDr-01B), a digital cotton-spinning frame (DSRo-04-2), and a digital spinning frame (DSSp-05B-S-C-G). The spinning process is as follows: open cleaning (preparation before carding)→carding→merging (head merging, two merging, three merging)→roving→spinning. Five different blending ratios (100/0, 80/20, 60/40, 40/60, and 20/80) with a fineness of 18.5 tex were designed and prepared by controlling the number of blending strips, roving, and spinning process parameters, which were labeled Y1, Y2, Y3, Y4, and Y5, respectively.

### 2.4. Weaving Process of Negative Ion Knitted Fabric

**Figure 2:**
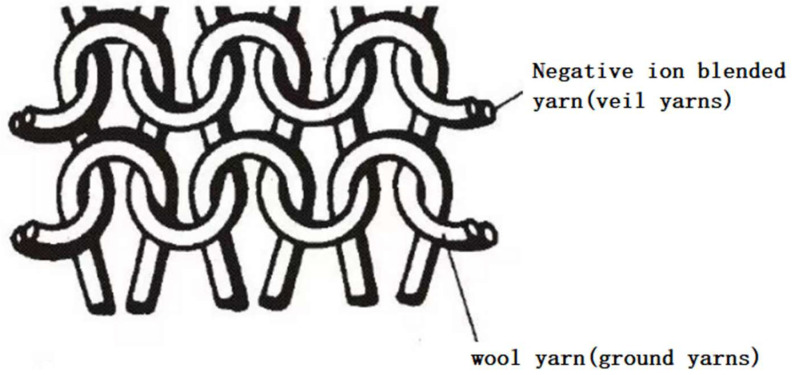
Schematic diagram of the knitted structure of sample A.

Double-sided knitted fabric is a kind of high-performance textile with a double or multilayer structure and various functions, both sides of which can be used. It usually has good wrinkle resistance, elasticity, and softness and is comfortable to wear and durable. It can achieve a variety of appearances and functions through different yarn combinations and occupies an important position in the textile market. In this part, two groups of different double-sided knitted fabrics were developed by presetting different yarn combinations and the structural parameters of the fabric. The specific raw material ratio and process of each group are described as follows:

(1) The blended yarns (Y1 and Y4) were selected as the veil and the wool yarns were selected as the ground yarns. After pretreatment, they were fed into a fully formed horizontal knitting machine (SWG061N2, Shimajima Seiki Co., Ltd., Kochi, Japan) according to the predetermined proportion, and the parameters of the machine such as needle distance, needle number, and drawing tension were set according to the preset specifications. The knitted structure of the specific sample is shown in Figure 2 and the specifications of the knitted fabric are shown in Table 3. Sample numbers are denoted as A1 and A2.

(2) Using negative ion polyester filament yarn and spandex yarn as the main raw materials and adjusting the filling ratio of weft yarn and the structural parameters of the fabric, a double-sided cylinder knitting machine (WDCJ, Quanzhou Bushuo Machinery Co., Ltd., Quanhou, China) was used to weave the double-sided knitted fabric sample of Group B. Details of the yarn ratio, organization structure, and equipment process are shown in Table 4. Sample numbers are denoted as B1 and B2.

Knitting key process: In the weaving process, it is necessary to pay more attention to the uniformity of the control tension and the coordination of the feeding speed, otherwise it will affect the consistency of the density of the grey cloth and then affect the later shaping and dyeing effect.

### 2.5. Test Methods

#### 2.5.1. SEM and EDS

Scanning electron microscopy with energy dispersive X-ray spectroscopy (ΣIGMA, ZEISS, Oberkochen, Germany) was used to analyze the elemental composition of the ZnO/TM composite and observe the morphology of the ZnO/TM composite and ZnO/TM/PET fiber. The samples were processed via gold sputtering with an accelerating voltage of 20 kV.

#### 2.5.2. Yarn Hairiness Index Testing

By means of a yarn hairiness tester (YG171B, Ningbo Textile Instrument Co., Ltd., Ningbo, China), the number, length, and distribution of hairiness on the yarn surface was measured and calculated using the projection counting method, and the hairiness index was subsequently obtained. The reference standard used was FZ/T 01086-2020 “Textile yarn hairiness determination method projection counting Method” [26].

#### 2.5.3. Wear Resistance Testing

The Martindale wear-resistant instrument (YG401H, Ningbo Textile Instrument Co., Ltd.) and a standard light source box (YG982, Ningbo Textile Instrument Co., Ltd) were used to evaluate the pining line of the samples. All samples had to be equilibrated under the specified standard atmospheric conditions (20 °C ± 2 °C, 65% ± 2% RH) for at least 24 h prior to testing. The sample size was a circular specimen with a diameter of about 38 mm. The friction cloth selected was standard wool felt. The reference standard was GB/T 4802.2-2008 “Determination of fabric propensity to surface fuzzing to pilling—Part 2: Modified Martindale method” [27].

#### 2.5.4. Mechanical Properties Testing

The mechanical properties, including breaking strength and breaking extension, of the samples were tested using a multifunctional electronic fabric strength machine (YG026D, Ningbo Textile Instrument Co., Ltd.). All samples had to be equilibrated under the specified standard atmospheric conditions (20 °C ± 2 °C, 65% ± 2% RH) for at least 24 h prior to testing. For the single yarn-breaking strength test, the sample length was 250 mm, and 30 samples were randomly intercepted for testing. For the fabric-breaking strength test, the sample size was 50 mm × 200 mm, and 5 strips were taken along the longitudinal and lateral aspects of the fabric. The spacing length was 100 mm and the drawing speed was 100 mm/min. The reference standard was GB/T 3923.1-2013 “Textiles—Determination of tensile properties of fabrics—Part 1: Breaking strength and elongation at break (strip method)” [28]. 

#### 2.5.5. Air Permeability and Moisture Permeability

The air permeability of the samples was tested using an automatic air permeability meter (YG461G-II, Wenzhou Darong Textile Instrument Co., Ltd., Wenzhou, China). The test pressure was 100 Pa, the nozzle diameter was 3.0 cm, and the test area was 20 cm^2^. The reference standard was GB/T 5453-1997 “Determination of air permeability of textile fabrics” [29].

The moisture permeability of fabric samples was tested using a computerized fabric permeator (YGB216T, Wenzhou Darong Textile Instrument Co., Ltd.). We adopted a type of standard washing machine, selected the 4 N program, selected the detergent as “standard detergent 3”, washed the fabric 3 times, and hung the samples to dry after each wash. The reference standard was GB/T 12704.1-2009 “Determination of moisture permeability of textile fabric” [30].

#### 2.5.6. Negative Ion Emission Testing

The negative ion emission of fabric samples was determined using a fabric negative ion generation tester DR407M, Wenzhou Darong Textile Instrument Co., Ltd.). The reference standards were “Detection and Evaluation of the occurrence of negative ions in Textiles” (GB/T 30128-2013) [31] and “Inspection methods for Import and Export Functional Textiles Part II: Negative Ion Content” (SN/T 2558.2-2011) [32].

#### 2.5.7. Far-Infrared Performance Testing

The far-infrared radiation temperature increase performances of the textile samples were measured using the textile far-infrared temperature rise rate tester (FFZ41 1-I, Wenzhou Fangyuan Instrument Co., Ltd., Wenzhou, China). The reference standard was GB/T 30127-2013 “Testing and Evaluation of far-infrared Properties of Textiles” [33]. The far-infrared emissivity of the fabric samples was tested by a far-infrared emissivity tester (EMS-302, Wenzhou Fangyuan Instrument Co., Ltd.). The reference standard was GB/T 30127-2013 “Testing and Evaluation of far-infrared Properties of Textiles”.

#### 2.5.8. Evaluation of Antimicrobial Properties

According to FZ/T 73023-2006 “Antimicrobial Knitwear Appendix D: Oscillating Method” [34], the samples of the prepared antimicrobial polyester knitted fabric were washed 50 times and 100 times, respectively. Then, the antimicrobial rate of the fabric against *Staphylococcus aureus* (ATCC6538), *Escherichia coli* (ATCC25922), and *Candida albicans* (ATCC10231) before and after washing was tested. The testing steps were as follows: first, we placed the sample to be tested into a culture solution containing bacteria or fungi at a certain concentration. Then, we placed the sample on a reciprocating shaker and shook it at a speed of 150 r/min for 18 h at a temperature of (24 ± 1) °C to ensure that the sample fabric was in full contact with the bacterial or fungal solution. Lastly, we cultivated the above-mentioned sample in a biochemical incubator at a temperature of (37 ± 1) °C for 24 h. We determined the number of bacterial or fungal colonies before and after shaking and quantitatively evaluated the antimicrobial effect using the colony-counting method. The method of calculation was as follows: Bacteriostasis rate (%) = (A − B)/A × 100%, where A is the number of colonies of the standard blank sample after shaking for 18 h and B is the number of colonies of the sample to be tested after shaking for 18 h. The antimicrobial durability of the sample of the antimicrobial polyester knitted fabric was evaluated according to FZ/T 73023-2006 “Antibacterial knitwear”.

## 3. Results and Discussion

### 3.1. SEM and EDS Analysis of ZnO/TM Composite and ZnO/TM/PET Fiber

**Figure 3:**
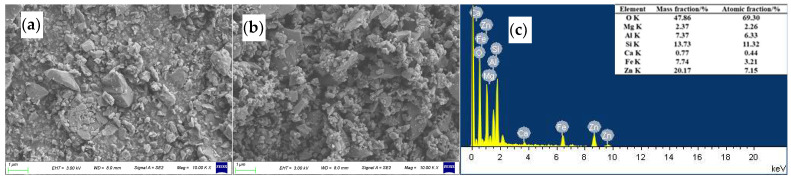
SEM and EDS of the sample: (**a**) raw tourmaline powder, (**b**) zinc oxide compound tourmaline powder, and (**c**) EDS atlas of the composite powder.

Figure 3 shows the micromorphologies of the ZnO/TM composite powder and tourmaline raw materials. As can be seen in Figure 3a,b, compared with unground tourmaline, the composite powder particles prepared via grinding are significantly reduced and the particle size is evenly dispersed, which is crucial for the preparation of functional fibers. It can be seen in Figure 3c that zinc oxide is effectively attached to the tourmaline surface during the ball-milling process, which ensures the good stability of the ZnO/TM composite powder. At the same time, the feasibility of the composite powder grinding process is verified again.

**Figure 4:**
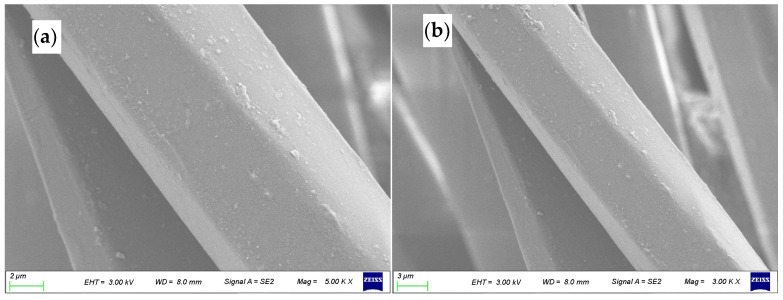
SEM of the ZnO/TM /PET fiber: (**a**) 5000×, (**b**) 3000×.

Figure 4 shows the longitudinal view of ZnO/TM/PET negative ion fiber under 5000× and 3000× magnification, from which ZnO/TM fillers are found to be distributed in the polymer matrix randomly without visible conglobation, which can be seen by comparing this figure with Figure 3b. Moreover, there is only a small quantity of ZnO/TM protruding slightly from the fiber surface. This homogeneous distribution is beneficial to negative ion fiber and contributes to its superior performance.

### 3.2. Effect of Blending Ratio on Properties of Negative Ion Functional Blended Yarn

#### 3.2.1. Hairiness Index Analysis

**Figure 5:**
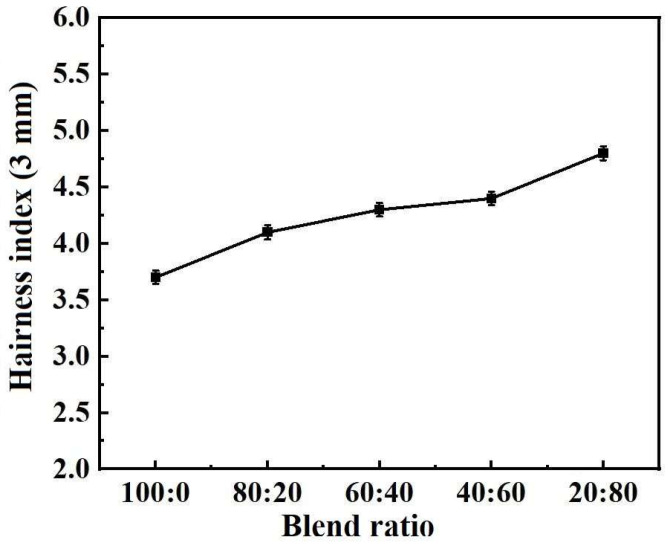
Hairiness index of five blended yarns with different raw material ratios.

Figure 5 shows the hairiness indexes of the five kinds of blended yarn samples. It can be seen in Figure 5 that with an increase in the proportion of cotton fiber, the hairiness index gradually increases. The addition of cotton fiber increases the hair on the surface of the yarn, reduces the smoothness of the yarn to a certain extent, and affects the appearance and feel of the yarn.

#### 3.2.2. Mechanical Property Analysis

**Figure 6:**
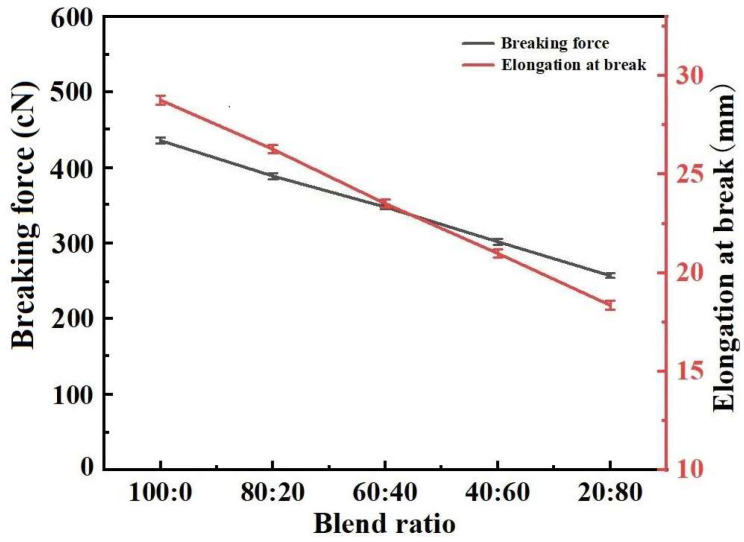
Mechanical properties of five blended yarns.

Figure 6 shows the mechanical properties of the blended yarn samples. As shown in Figure 5, with an increase in the proportion of cotton fiber in the blend, the related indexes of the yarn’s mechanical properties gradually decline. This is due to the differences in mechanical properties between polyester fiber and cotton fiber. When the external force stretches the blended yarn, the two jointly bear the tension at the beginning stage. As the duration of stretching increases, the cotton fiber will break first due to its poor elasticity. With the further intensification of stretching, the negative ion polyester fiber, with its good tensile strength and extensibility, mainly bears increasing tension until the blended yarn breaks completely.

#### 3.2.3. Negative Ion Release Analysis

**Figure 7:**
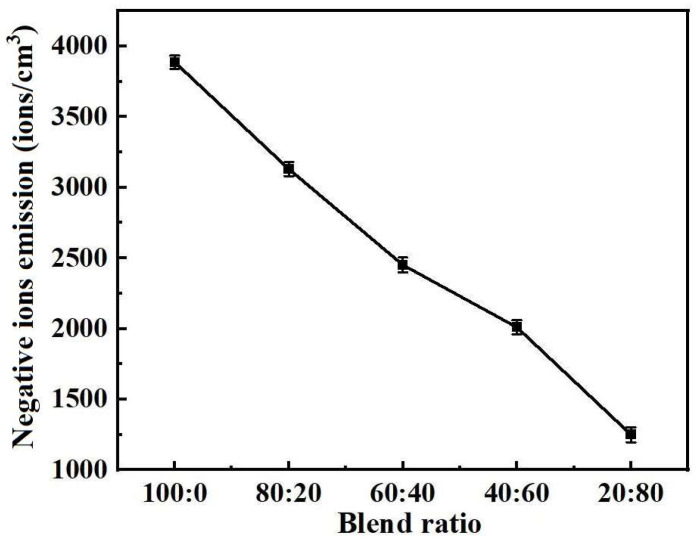
Negative ion emission of five blended yarn samples.

Figure 7 shows the negative ion emissions of the five kinds of blended yarn samples. As can be seen in Figure 7, the negative ion emission of the sample decreases with the decrease in the proportion of negative ion polyester fiber in the blended yarn. The negative ion emission performance of the blended yarn mainly depends on the negative ion polyester fiber, and its ratio has an important effect on the negative ion release ability of the blended yarn. In practical applications, a suitable blending ratio can be selected according to specific needs to balance appearance properties, mechanical properties, and functional characteristics to obtain ideal yarn products.

### 3.3. Effect of Yarn Ratio and Fabric Structure on Properties of Double-Sided Knitted Fabric with Negative Ion Function

#### 3.3.1. Mechanical Properties Analysis

Table 5 shows the mechanical properties of the fabric samples. As shown in Table 5, the mechanical properties of group B fabrics are better than those of group A, showing better stability and ductility. The mechanical properties of fabrics are closely related to the types of yarn selected and the fabric structure. From the analysis of fabric-weaving materials, the fabric in group A is made of negative ion polyester/cotton-blended yarn and wool yarn, while the fabric in group B is made of negative ion polyester filament and spandex. The breaking strength of negative ion polyester filament yarn is higher than that of negative ion polyester/cotton-blended yarn and wool yarn. Combined with the excellent elasticity of spandex yarn, this supports the excellent performance of the fabrics in group B. From the analysis of the structure, the fabrics in group A are made of low-density double-sided thread, which makes the fabric loose and soft to the hand with strong fluff on the surface of the fabric. The fabrics in group B are made of tight double-sided thread, which not only has a smooth surface and no curling but also has little dispersion. The fabric with the double-thread organization can only be extended to the reverse knitting direction, even if the thread is separated. This may be another reason why the mechanical properties of the former are better than those of the latter.

In addition, upon comparing and analyzing fabric samples A1 and A2, it is not difficult to see in Table 5 that the mechanical property index of fabric A1 is significantly higher than that of A2, which is mainly related to the yarn used in the fabric. The ground yarns of fabrics A1 and A2 are wool yarn, but the veils of the two are different. The veil used in fabric A1 is pure negative ion polyester staple yarn, while the veil used in fabric A2 is 40/60 negative ion polyester/cotton blend yarn. The mechanical properties of pure negative ion polyester fiber are better than those of blended yarn. Therefore, the mechanical properties of fabric A1 are significantly stronger than those of A2. Upon comparing the mechanical indexes of fabrics B1 and B2, it can be seen in Table 5 that the longitudinal breaking strength of B1 is lower than B2 and the breaking elongation of B1 is better than B2. This is because, compared with fabric B2, the proportion of negative ion polyester yarn with higher breaking strength in fabric B1 is relatively low, resulting in lower longitudinal breaking strength. Meanwhile, the proportion of spandex yarn with better elasticity in fabric B2 is relatively high, which results in a higher breaking elongation index for fabric B2. Therefore, the mechanical properties of the fabric can be adjusted by adjusting the yarn ratio and fabric structure to meet the application requirements of different occasions.

#### 3.3.2. Pilling Resistance Analysis

The evaluation of the pilling resistance of our fabric samples is shown in Table 6. As can be seen in Table 6, all four fabrics show higher wear resistance, and the wear resistance of fabric B is better than that of the A group. This is related to the fabric structure and raw materials. The double-sided knitted fabric itself has better elasticity and morphological stability, coupled with the superior characteristics of wool yarn and spandex yarn, which reduces the chance of fabric resistance to friction damage. In terms of the raw materials, those of group B fabric are negative ion polyester filament yarn and spandex yarn, while the raw materials of group A are negative ion polyester/cotton-blended yarn and wool yarn. The tensile breaking strength and elasticity of the former are better than those of the latter. In terms of the organizational structure, the tightness of group A is lower than that of group B. These two factors contribute to the pilling performance of group B being better than that of group A. It can also be seen in Table 6 that the pilling score decreased after more iterations of friction. This is in line with our expectations because more iterations of friction lead to greater surface damage of the fabric and more serious phenomena of fuzz and pilling.

#### 3.3.3. Air Permeability and Moisture Permeability Analysis

**Figure 8:**
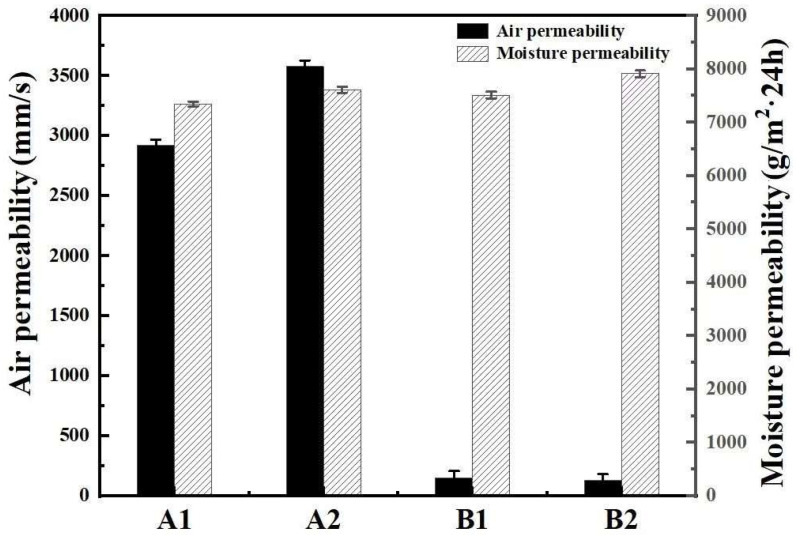
Air permeability and moisture permeability of the two double-sided knitted fabric samples.

Figure 8 shows the evaluation of the air permeability and moisture permeability of the fabric samples. The air permeability and moisture permeability of fabrics are important indicators to measure their comfort and functionality, especially in sportswear, outdoor clothing, and functional textiles. Air permeability refers to the ability of air to pass through the fabric. Fabrics with good air permeability can allow air to circulate freely and improve wearing comfort, especially in hot or high-intensity activities. The air permeability of fabric is related to the fabric density, fiber type, and fabric structure, especially fabric density [35]. The less tight the fabric is, the less difficult it is for air to pass through and the better the permeability. It can be seen in Figure 8 that the air permeability of group A is significantly better than that of group B. The main reason is that the fabric structure of group A is relatively loose, while the fabric structure of group B is relatively tight. Moisture permeability refers to the ability of water vapor to pass through the fabric. Fabric with good moisture permeability can quickly evaporate human sweat and keep the skin dry, making it suitable for sports, outdoors, and other scenes. The moisture permeability of fabric is the result of the fiber hygroscopicity, fabric structure, and other factors. It can be seen in Figure 5 that the moisture permeability of the two group samples is more than 7000 g/(m^2^·24 h). According to the standard of GB/T41788-2022 “Multi-functional knitted products” [36], the moisture permeability (before and after washing) of knitted products with moisture permeability function is ≥2200 g/(m^2^·24 h). It is easy to see that the moisture permeability of the two groups of samples far exceeds these standard requirements.

#### 3.3.4. Negative Ion Release Property

**Figure 9:**
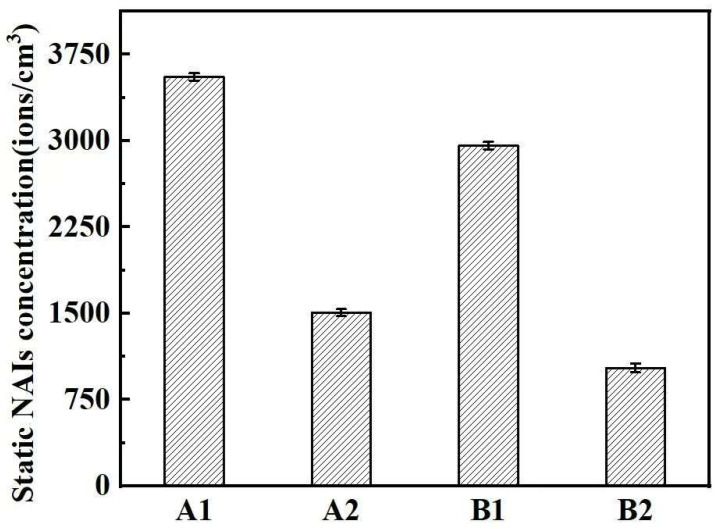
Negative ion release properties of the two groups of fabric samples.

Figure 9 shows the negative ion emissions released from the samples. As can be seen in Figure 9, the amount of negative ions released from the samples of both groups of fabric is more than 1000 /cm^3^. According to the evaluation description of “negative ion release amount” in GB/T 30128-2013 “ Textiles—Testing and evaluation for negative-ion concentration”, a negative ion emission amount >1000 /m^3^ is identified as high negative ion emissions. Therefore, it can be considered that the two groups of fabric samples have high negative ion release performances. Comparing the A1 and A2 fabrics, it can be seen that the negative ion emission of the A1 fabric is higher than that of A2. Comparing B1 and B2 fabrics, it can be seen that the negative ion emission of fabric B1 is higher than B2. This is related to the ratio of negative ion polyester fiber in the fabric and its structure. In terms of the fiber ratio, under the premise of the same fabric structure and fiber composition, the higher the ratio of negative oxygen ion polyester fiber, the higher the negative ion emissions of the fabric. This is the reason for the order of A1 > A2, B1 > B2 regarding the release of negative ions. In addition, in terms of the fabric structure, the loose structure of the fabric facilitates sliding and friction between the fibers. The negative ion function of the fiber mainly comes from the ionization effect of the dispersed tourmaline powder blended in fiber on the water in the air. When the fabric is subjected to external pressure, the yarn of the loose-structure fabric makes it easier to slip and make contact with the air, which helps to enhance the negative ion release performance of the fiber in the fabric. Therefore, compared with the tight fabric structure, the loose structure is more conducive to the sliding and friction of the fiber.

#### 3.3.5. Far-Infrared Performance

**Figure 10:**
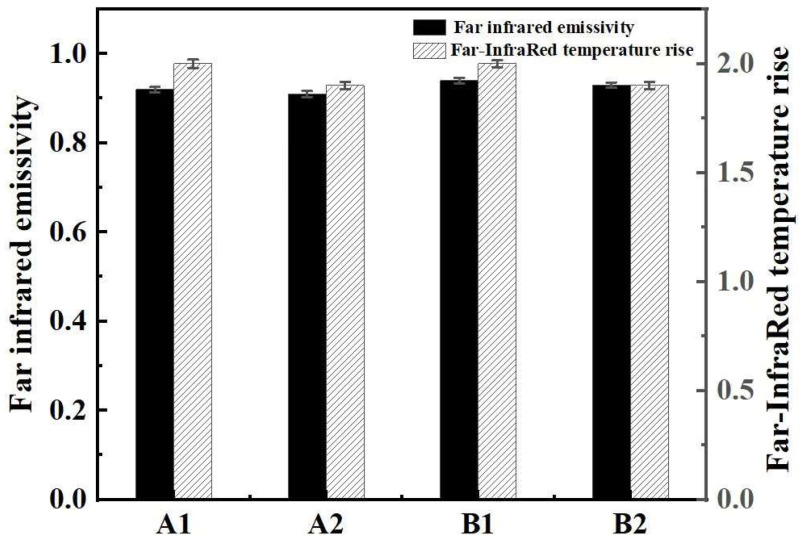
Far-infrared properties of the two groups of fabric samples.

Figure 10 shows the far-infrared properties of the fabric samples. As can be seen in Figure 10, the far-infrared radiation heating index of the two groups of negative ion functional fabric samples was higher than 0.91, and the far-infrared emission index reached 1.9 °C. According to the evaluation criteria for far-infrared emission performances of fabrics in GB/T 30127-2013 “Detection and Evaluation of Far infrared Properties of Textiles”, the radiation temperature increase in far-infrared textiles should be ≥1.4 °C, and the far-infrared emissivity should be ≥0.88. Therefore, it can be considered that the four fabric samples are categorized as far-infrared functional textiles. The far-infrared emission properties of these four fabrics are mainly from the negative ion functional fibers. The negative ion additive of negative ion polyester fiber is tourmaline, which is the source of far-infrared emissions. The mechanism of the far-infrared emissions of tourmaline is the lattice vibration triggered by thermoelectric and piezoelectric effects [37], which is further enhanced by their unique crystal structure and chemical composition. When subjected to a temperature change (such as heating or cooling) or an external force, the positive and negative charge centers inside the tourmaline crystal will shift relative to each other, resulting in instantaneous voltage on the surface of the material. This thermoelectric effect causes the charged particles (such as ions or polar molecules) in the lattice to vibrate. The frequencies of these vibrations were in the far-infrared band (5.6–1000 μm). According to electromagnetic theory, the charge of vibration radiates electromagnetic waves, so tourmaline converts thermal energy into far-infrared radiation through the thermoelectric effect. Metal ions such as Fe^2^⁺/Fe^3^⁺ and Mg^2^⁺ in tourmaline may be involved in the electronic transition or local vibration to generate far-infrared radiation of a specific wavelength [38]. The high bond strength and vibrational mode of B-O bonds may contribute to the far-infrared radiation at high-frequency bands. This performance characteristic of negative ion functional double-sided knitted fabrics may make them a good application prospect in the development of products for keeping warm and promoting blood circulation.

#### 3.3.6. Antimicrobial Performance

Table 7 and Table 8 show the test results of the antimicrobial properties of the four fabric samples. As can be seen in Table 7, after washing the fabrics 50 times, the inhibition rates of the two groups of fabric samples against *Staphylococcus aureus*, *Escherichia coli,* and *Candida albicans* are higher than the national standard requirements (FZ/T 73023-2006 “Appendix D, Oscillation method” and GB/T 20944.3-2008 “Evaluation of antimicrobial properties of textiles—Part 3: Oscillation method” [39]), showing good antimicrobial activity. The antimicrobial properties of negative ion functional fabric mainly come from the negative ion functional yarn. The raw material for fiber preparation is ZnO/TM composite powder, which has an obvious inhibitory effect on bacteria and fungi [25]. Moreover, the antimicrobial property of negative ion functional fabric is affected by the proportion of negative ion fiber in the fabric, which shows that the higher the content of negative ion fiber yarn, the better the antimicrobial properties. Comparing the antimicrobial properties of the two groups of fabric samples, it is not difficult to see that the antimicrobial properties of fabric A1 is better than that of A2 and that of fabric B1 is better than that of B2. In addition, it can also be seen in Table 7 that the antimicrobial properties of the two groups of fabric samples are still very good after 100 washes, which proves that the negative ion functional fiber has excellent antimicrobial properties and great market application potential in the development of antimicrobial products.

## 4. Conclusions

Five kinds of negative ion polyester/cotton blended yarn were spun with negative ion functional polyester fiber, cotton fiber, wool yarn, and spandex yarn as the main raw materials, and four different kinds of knitted double-sided fabrics were developed. The effects of the blending ratio on the performance of blended yarn and the yarn ratio and fabric structure on fabric wear and functional characteristics were studied, and the following conclusions were obtained:

1. The effects of the blending ratio on the performance of functional polyester/cotton blended yarn are significant. With the increase in the ratio of negative ion fiber in blended yarn, the mechanical properties and negative ion release of the blended yarn were significantly enhanced, and the hairiness index decreased.

2. The performances of functional fabric are affected by the fiber raw material type, yarn ratio, fabric tightness, and organizational structure. (1) Under the same structural parameters, fabrics with high negative ion polyester fiber content have relatively strong negative ion emission, good far-infrared emission performances, and antibacterial properties. (2) Under the same yarn ratio, the negative ion emission performance of the fabric with a loose structure is better than that of the fabric with a tight structure, but the differences in moisture permeability across the four fabrics are not obvious. (3) Purely from the point of view of mechanical properties, the knitted double-sided fabric made of negative ion polyester filament/spandex is superior to the double-sided fabric made of negative ion polyester/cotton-blended yarn and woolen yarn.

Compared with relative textile product standards, these four functional double-sided knitted fabric samples showed excellent wear performance and functional characteristics, which can provide a reference for its application. This study is only an application attempt for negative ion functional fiber and yarn, and future research should further develop and experiment with fabrics that have different structures to provide references for the application and promotion of high-performance polymers.

## Figures and Tables

**Table 1:** Temperature of each zone of melt spinning equipment.

Process Condition	1st Area Screw Temp.	2nd Area Screw Temp.	3rd Area Screw Temp.	4th Area Screw Temp.	Screw Flange Temp.	Metering Pump Temp.	1st Area Hot Roll Temp.	2nd Area Hot Roll Temp.	3rd Area Hot Roll Temp.
SV(Actual temp) (°C)	202	260	270	270	269.9	265.2	65.1	66.3	68.2
PV(Set temp) (°C)	200	260	270	270	270.0	65.0	65.0	65.0	65.0

**Table 2:** Spinning process parameters.

Process Condition	Extruder Screw Frequency (Hz)	Extrusion Screw Current (A)	Metering Pump Speed (r/min)	Oiling Speed (r/min)	1st Area Hot Roll Speed (r/min)	2nd Area Hot Roll Speed (r/min)	3rd Area Hot Roll Speed (r/min)	Winding Speed (r/min)	Winding Angle (°)	Theoretical Drawing Ratio
Process parameter	2.00	8.90	5.0	3.0	440.0	700.0	760.0	800.0	5.0	1.8

**Table 3:** Preset specification parameters for group A.

Fabric Sample	Yarn Combination	Yarn Ratio (%)	Square Meter Weight (g/m^2^)	Number of Stitches on the Front of the Fabric (Stitches/5 cm)		Number of Stitches on the Back of the Fabric (Stitches/5 cm)	
				Transverse Density	Longitudinal Density	Transverse Density	Longitudinal Density
A1	wool/Y1	50:50	250	30	40	30	40
A2	wool/Y4	50:50	250	30	40	30	40

**Table 4:** Process parameters of the double-sided knitted fabric sample of group B.

Fabric Sample	Yarn Ratio (%)		Equipment Operating Parameter			
	ZnO/TM/PET Yarn	Spandex Yarn	Feeder (F)	Intube Diameter (Inch)	Machine Gauge (Needles)	Machine Speed (r/min)
B1	78	22	84	34	28	25
B2	87	13	84	34	28	25

**Table 5:** Mechanical properties of two kinds of double-sided knitted fabric samples.

Fabric Sample	Breaking Strength (N)		Breaking Extension (mm)	
	Weft	Warp	Weft	Warp
A1	310.26	251.17	58.41	137.69
A2	168.17	126.43	40.01	67.84
B1	311.02	419.94	390.24	276.26
B2	353.62	393.86	319.01	210.92

**Table 6:** The pilling resistance of the two groups of double-sided knitted fabric samples.

Number of Frictions		500		1000		2000		5000		7000	
Test Item		Fuzzing	Pilling	Fuzzing	Pilling	Fuzzing	Pilling	Fuzzing	Pilling	Fuzzing	Pilling
A1	Right Side	5	5	4.5	4.9	4.3	4.6	4	4.2	3.8	4
	Reverse Side	5	5	4.5	4.9	4.3	4.6	4	4.2	3.8	4
A2	Right Side	5	5	4.5	4.9	4.3	4.6	4	4.2	3.8	4
	Reverse Side	5	5	4.5	4.9	4.3	4.6	4	4.2	3.8	4
B1	Right Side	5	5	4.8	5	4.5	4.9	4.2	4.6	4	4.2
	Reverse Side	5	5	4.8	5	4.5	4.9	4.2	4.6	4	4.2
B2	Right Side	5	5	4.8	5	4.5	4.9	4.2	4.6	4	4.2
	Reverse Side	5	5	4.8	5	4.5	4.9	4.2	4.6	4	4.2

**Table 7:** Antibacterial test indexes of the two groups of fabric samples.

Microbial species		*Staphylococcus aureus*		*Escherichia coli*		*Candida albicans*	
Number of washes/times		50	100	50	100	50	100
Antimicrobial Rate (%)	Standard requirement	≥80	≥90	≥70	≥80	≥60	≥70
	A1	98	95	95	91	85	81
	A2	97	94	95	91	85	81
	B1	98	95	95	91	85	81
	B2	97	94	95	91	85	81
Evaluation result		antimicrobial					

**Table 8:** Digital photos of the plane count results of *Staphylococcus aureus*, *Escherichia coli*, and *Candida albicans* for control sample and fabric samples A1 and B1.

Microbial Name	Control Sample	Sample A1		Control Sample	Sample B1	
		Before Washed	After Washed 50 Times		Before Washed	After Washed 50 times
*Staphylococcus aureus*	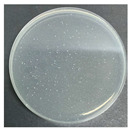	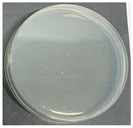	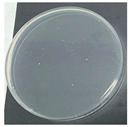	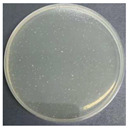	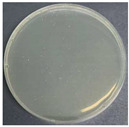	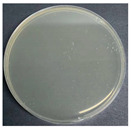
*Escherichia coli*	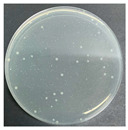	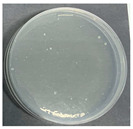	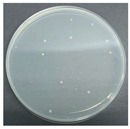	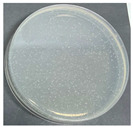	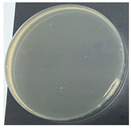	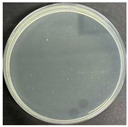
*Candida albicans*	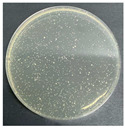	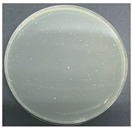	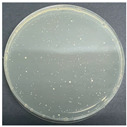	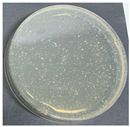	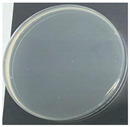	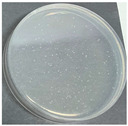

## Data Availability

The original contributions presented in this study are included in the article. Further inquiries can be directed to the corresponding author.
